# Long-Term Medical Resource Consumption of Radical Prostatectomy vs. Intensity-Modulated Radiotherapy for Old Patients With Prostate Cancer: A Nationwide Population-Based Cohort Study

**DOI:** 10.3389/fmed.2022.843709

**Published:** 2022-05-03

**Authors:** Szu-Yuan Wu, Fransisca Fortunata Effendi, Jhao Yang Peng, Chung-Chien Huang

**Affiliations:** ^1^Department of Food Nutrition and Health Biotechnology, College of Medical and Health Science, Asia University, Taichung, Taiwan; ^2^Big Data Center, Lo-Hsu Medical Foundation, Lotung Poh-Ai Hospital, Yilan, Taiwan; ^3^Division of Radiation Oncology, Lo-Hsu Medical Foundation, Lotung Poh-Ai Hospital, Yilan, Taiwan; ^4^Department of Healthcare Administration, College of Medical and Health Science, Asia University, Taichung, Taiwan; ^5^Cancer Center, Lo-Hsu Medical Foundation, Lotung Poh-Ai Hospital, Yilan, Taiwan; ^6^Graduate Institute of Business Administration, Fu Jen Catholic University, Taipei, Taiwan; ^7^Centers for Regional Anesthesia and Pain Medicine, Taipei Municipal Wan Fang Hospital, Taipei Medical University, Taipei, Taiwan; ^8^Master Program in School of Health Care Administration, Department of Health Care Administration, College of Management, Taipei Medical University, Taipei, Taiwan; ^9^PT Inertia Utama, Dexa Group, South Tangerang, Indonesia; ^10^Graduate Institute of Business Administration, College of Management, Fu Jen Catholic University, New Taipei City, Taiwan; ^11^Roche Diagnostics Ltd., New Taipei City, Taiwan; ^12^International Ph.D. Program in Biotech and Healthcare Management, School of Health Care Administration, College of Management, Taipei Medical University, Taipei, Taiwan; ^13^Department of Medical Quality, Taipei Municipal Wan Fang Hospital-Managed by Taipei Medical University, Taipei, Taiwan

**Keywords:** medical resource consumption, radical prostatectomy, intensity-modulated radiation therapy, old-age, localized prostate cancer

## Abstract

**Purpose:**

Few studies have compared the long-term medical resource consumption between radical prostatectomy (RP) and intensity-modulated radiation therapy (IMRT) among old (≥80 years) patients with localized prostate cancer (LPC), particularly in those at high risk of prostate adenocarcinoma.

**Patients and Methods:**

The propensity score matching was conducted to investigate the medical expenditure of two therapeutic modalities (RP and IMRT) in elderly patients with high-risk LPC (HR-LPC). The generalized linear mixed and logistic regression models were employed to evaluate the number of postdischarge visits and medical reimbursement for urinary diseases or complications and the number of hospitalizations for treatment-related complications over 5 years after treatment, respectively.

**Results:**

Significant differences were observed in the median or mean urology clinic visit numbers across the two therapeutic modalities from the first until fifth year post treatment (*p* < 0.0001). After adjustment for covariates, the mean difference [95% confidence interval (CI)] of urology clinic visit numbers between RP and IMRT was 13.07 (10.45–15.49, *P* < 0.0001), 7.47 (8.01–14.92, *P* < 0.0001), 8.24 (4.59–9.90, *P* < 0.0001), 6.63 (3.55–11.70, *P* < 0.0001), and 5.02 (1.12–8.73, *P* < 0.0001) for the first, second, third, fourth, and fifth years, respectively. In the logistic regression multivariate model with adjustment for covariates [therapy type, age, diagnosis year, income, hospital area, hospital level (academic or nonacademic), clinical and pathological T-stage, grade (Gleason score), pretreatment PSA level (ng/ml), and D'Amico risk classification], the adjusted odds ratio (95% CI) of IMRT was 2.10 (1.37–2.56, *P* = 0.0013), 1.55 (1.08–2.21, *P* = 0.0151), 1.35 (1.08–2.21, *P* = 0.0084), 1.24 (1.07–2.21, *P* = 0.0071), and 1.09 (1.02–1.81, *P* = 0.0379) for the first, second, third, fourth, and fifth years, respectively, compared with those of RP. The mean difference (95% CI) of total medical claims amounts of RP and IMRT between the RP and IMRT + ADT groups was 2,69,823 New Taiwan Dollars (NTD) (247,676–291,970, *P* < 0.0001), 40,803 NTD (17,379–54,228, *P* < 0.0001), 36,202 NTD (24,375–68,029, *P* < 0.0001), 26,708 NTD (11,179–54,595, *P* = 0.0321), and 12,173 NTD (17,140–41,487, *P* = 0.0187) for the first, second, third, fourth, and fifth years, respectively.

**Conclusion:**

The long-term medical resource consumption was higher in old men with HR-LPC undergoing IMRT than in those undergoing RP.

## Introduction

Localized prostate cancer (LPC) mean prostate cancer is still confined within prostate glands without extension to other sites in the patients. LPC is commonly asymptomatic if it has been diagnosed in the early stage, because slowly progression of disease (1, 2). Consequently, fewer older men receive curative-intent therapy, namely radiotherapy (RT) or radical prostatectomy (RP), compared with younger men, because elderly patients with LPC might receive conservative treatments (1). Active surveillance is generally the treatment strategy applied in older men (2). Between the aforementioned two curative-intent therapies, RT is preferable for older men, who are typically aged more than 70 years, (1, 3, 4) whereas another therapy, such as watchful waiting or androgen deprivation therapy (ADT) with luteinizing hormone-releasing hormone agonist, are preferable for men older than 80 years (1). In Taiwan, the most common risk classification used is the National Comprehensive Cancer Network (NCCN) risk classification depending on the clinical tumor (T) stage; Gleason scores and Pretreatment Prostate-Specific Antigen (PSA) are applied for further decision-making based on NCCN guidelines (5). Even for old (≥80 years) men with NCCN high-risk LPC (NCCN-HR-LPC) with a life expectancy of >5 years, more aggressive treatments such as RT or RP are suggested as per NCCN guidelines (5). understanding the medical resource consumption of the two curative treatments is valuable for establishing health policies, and the results can be used as a reference for implementing relevant national health services.

The treatment of patients with PC is expensive (3). Studies have provided inconsistent results regarding the cost of RP and RT (3, 6, 7). Some studies have shown that the expenditure incurred in RP is higher than that incurred in RT (3), which was most likely caused by the emergence of the advanced RP techniques, namely laparoscopic RP and robot-assisted radical prostatectomy (RARP) (8). In addition, the hospitalization cost of RP is significantly higher than that of RT, as the major proportion of RT patients are outpatients (3). A study evaluated the value of RP based on the morbidity and mortality rates and found that overall adjusted in-hospital mortality after radical prostatectomy was relatively low (0.25%), with a decreased length of hospitalization (6). Intensity-modulated radiation therapy (IMRT) is the contemporary RT technique; it is more suitable for HR-LPC, with a higher radiation dosage, higher dose conformity to cancer, and less radiation to normal tissues (9–13). RT with IMRT technique is more costly than the RT techniques applied in the studies that identified RP as more expensive than RT (7, 14). The medical resource consumption of RP and IMRT for men with NCCN-HR-LPC is unclear (3, 7, 14), especially in elderly patients. However, no long-term evaluation with a follow-up duration of >5 years has been conducted for the medical resource consumption of RP and high-dose IMRT plus long-term ADT in old men with NCCN-HR-LPC.

Geriatric medicine has gained increasing importance for cancer treatment because the average life span is increasing (15). The two curative-intent modalities of RP and IMRT are effective for improving the survival of old men with NCCN-HR-LPC (16–22), but no comparative study has been conducted for the medical resource consumption of the two treatments. This research gap leads to difficulty in shared decision-making between old patients and physicians. Therefore, we conducted this comparative study of the medical resource consumption of RP and high-dose IMRT with long-term ADT using propensity score matching (PSM) among old (≥80 years) men with NCCN-HR-LPC. The results would provide a valuable reference for shared decision-making between old patients and physicians in the future. Selection of the same clinical outcomes therapeutic option with less financial toxicity on patients and Taiwan's healthcare financing system would be important to establish the more cost-effective health policy in the near future, because Taiwan's health care system on the verge of collapse (23).

## Patients and Methods

### Study Cohort

The data were collected retrospectively from the Taiwan Cancer Registry Database (TCRD) and the Taiwan National Health Insurance (NHI) Research Database (NHIRD). All medical costs have been paid by NHI and data recorded in the NHIRD. The index date was the date of PC diagnosis. The cohort included patients aged ≥80 years who had been diagnosed with LPC and who had received RP or high-dose IMRT and long-term ADT between January 1, 2011, and December 31, 2016.

In the inclusion criteria, (1) RP was defined as surgical procedures to remove the entire prostate gland and its surrounding lymph nodes for men with LPC (24). (2) High-dose IMRT was defined for RT administered a 54 Gy to the seminal vesicles as well as cone-down boosts of 72–81 Gy to cover the prostate in 1.8 Gy per fraction. (3) Patients were confirmed through a review of following information: pathological data, magnetic resonance imaging for PC stratification (cT1-T3a), pretreatment PSA levels (>20 ng/ml), and grade based on GS ≥8. (4) According to the aforementioned criteria, patients were included in our cohort and were defined as having NCCN-HR-LPC (5). RP and high-dose (≥72 Gy) IMRT with long-term (≥18 months) adjuvant ADT were included as the curative-intent therapies for men with NCCN-HR-LPC and a life expectancy of >5 years.

In the exclusion criteria, (1) patients who had other cancers, clinical lymph node metastasis, or distant metastasis [based on the staging system of the American Joint Committee on Cancer (AJCC), 7^th^ edition] were excluded from this study. (2) Inadequate doses of IMRT (<72 Gy) were excluded from this study. (3) Patients with unidentified clinical or pathological stage, unidentified D'Amico risk classification, unidentified Gleason score, unidentified postoperative Gleason grade, missing data on pretreatment PSA levels, and nonadenocarcinoma histology were excluded from this study.

Furthermore, the comparison of the two procedures, were specified into the RP and high-dose IMRT plus long-term ADT groups, respectively. The follow-up duration was 5 years after the index date; the medical resource consumption of the two curative-intent therapies was calculated over these 5 years. The study protocols were reviewed and approved by the Institutional Review Board of Tzu-Chi Medical Foundation (IRB109-015-B).

### Prospensity Score Matching

To improve analysis precision, we employed head-to-head PSM between the RP and high-dose IMRT plus long-term ADT groups (25). Most of the independent variables were matched at a ratio of 1:2; the other variables were matched at a ratio of 1:2 or 1:1. To reduce the effects of potential confounders when comparing all-cause death between the RP and high-dose IMRT+ ADT groups, the participants were matched based on propensity scores. The matching variables used were age, year of diagnosis, CCI scores, myocardial infarction, congestive heart failure, peripheral vascular disease, cerebrovascular disease, chronic pulmonary disease, diabetes, hypertension, income levels, hospital areas, hospital levels (academic or non-academic hospitals), clinical T-stage, Gleason score, Grade (max of Gleason grade), preoperative PSA (ng/ml), and D'Amico classification. Comorbidities were determined according to ICD-9-CM codes in the main diagnosis of inpatient records or if the number of outpatient visits was ≥2 within 1 year. Continuous variables are presented as means ± standard deviations or medians (first and third quartiles), as appropriate. We matched the participants at a ratio of 1:1 or 1:2 by using the greedy method, matched with a propensity score within a caliper of 0.2 (26). Matching is a common technique for selecting controls with identical background covariates as study participants, and it is done to minimize differences among study participants (that the investigator deems necessary to be controlled for).

### Covariates and Endpoints

The primary independent variables in this study were RP and IMRT. The covariates were therapy type, age, diagnosis year, income, hospital area, hospital level (academic or nonacademic), clinical and pathological T-stage, grade (Gleason score), pretreatment PSA level (ng/ml), and D'Amico risk classification, which might be correlated with all-cause mortality. Comorbidities were evaluated using the Charlson comorbidity index (CCI) (27, 28). Comorbidities that were correlated with all-cause death and which occurred 6 months prior the index date were examined in this study. Comorbidities were identified based on International Classification of Diseases, Ninth Revision, Clinical Modification (ICD-9-CM) diagnostic codes; comorbidities were defined as those with more than two repetitive primary diagnostic codes for visits to the outpatient department or the first admission. The dependent variables were as follows: (1) the number of urology outpatient clinic visits, (2) the proportion of patients being hospitalized for urinary diseases or treatment-related complications, and (3) medical reimbursement for urinary diseases or treatment-related complications.

### Statistical Analysis

In this nationwide population-based cohort study, the generalized linear mixed model with multivariate analysis, with adjustment for covariates including age, clinical and pathological T-stage, Gleason score, preoperative PSA, D'Amico risk classification, hospital level, and therapeutic modality, was applied to compare the RP and high-dose IMRT+ ADT groups. The generalized linear mixed model fitted with the random intercept was used for grouping patients by the hospital level, and Type III tests of fixed effects were conducted. As a result, the *p*-value was the only indicator that could be observed. Descriptive statistics were used to describe patient characteristics based on the therapeutic modality. The descriptive statistics were the mean and standard deviation for normal continuous data, median and interquartile range for nonnormal continuous data, and number and proportion for categorical data. Student's *t* test, analysis of variance, and nonparametric counterpart tests were applied, as appropriate. Two types of multivariate mixed models stratified by the hospital level were fitted to ensure the effect of therapeutic modalities on the outcomes: (1) a linear model for continuous outcomes, number of urology outpatient clinic visits, and medical costs for therapeutic complications and (2) a logistic regression model for the number of hospitalizations for therapeutic complications, with adjustment for covariates. The significance level was set at 5%.

## Results

### Clinicopathological Characteristics

Totally, 659 patients were included in this study, comprising 277 and 382 who underwent RP and IMRT + ADT, respectively. Patients who underwent RP and IMRT + ADT were followed up for a mean period of 61.7 [standard deviation (SD) = 18.9] months and 58.4 (SD = 18.4) months, respectively. No statistically significant differences were observed in age, diagnosis year, CCI score, clinical T-stage, T-stage, postoperative Gleason score, Gleason grade, pretreatment PSA level, D'Amico risk classification, hospital level and area, follow-up duration, and income (Supplementary Table 1, online only).

### Number of Urology Outpatient Clinic Visits Stratified by RP and IMRT

Table 1 presents the number of urology outpatient clinic visits per patient classified by treatment approaches (RP and IMRT). Significant differences were observed in the median or mean urology clinic visit numbers across the two therapeutic modalities from the first until fifth year post treatment (*p* < 0.0001). The numbers of urology outpatient clinic visits per patient were significantly more in the IMRT group than in the RP group (Table 1). In the generalized linear mixed model with adjustment for covariates (Supplementary Table 1, online only), the mean difference [95% confidence interval (CI)] of RP and IMRT + ADT was 13.07 (10.45–15.49), 7.47 (8.01–14.92), 8.24 (4.59–9.90), 6.63 (3.55–11.70), and 5.02 (1.12–8.73) for the first, second, third, fourth, and fifth years, respectively, with all *p*-values of <0.0001. The median and mean clinic visit numbers significantly reduced from the initial year post treatment (median of 31 and 42 visits for RP and IMRT, respectively, *p* < 0.0001) to the latest follow-up in the fifth year (median of 20 and 22 visits for RP and IMRT, respectively, *p* < 0.0001).

**Table 1:** Generalized linear mixed model of numbers of urology outpatient clinic visits stratified by RP and IMRT.

**Numbers of outpatient clinic visits**		**RP (Ref) *N* = 277**	**IMRT *N* = 382**	Mean difference (95% CI)*	***p*-value**
		***N*, %**	***N*, %**		
First year after treatment	Mean (SD)	31.7 (12.9)	44.8 (16.8)	13.07 (10.45, 15.49)	<0.0001
	Median (IQR, Q1–Q3)	31 (22–39)	42 (32–53)		
Second year after treatment	Mean (SD)	28.2 (14.6)	35.7 (18.8)	7.47 (8.01, 14.92)	<0.0001
	Median (IQR, Q1–Q3)	25 (18–35)	33 (23–46)		
Third year after treatment	Mean (SD)	27.8 (15.3)	35.0 (18.7)	8.24 (4.59, 9.90)	<0.0001
	Median (IQR, Q1–Q3)	26 (17–35)	32 (21–44)		
Fourth year after treatment	Mean (SD)	24.5 (14.3)	30.7 (20.8)	6.63 (3.55, 11.70)	<0.0001
	Median (IQR, Q1–Q3)	23 (12–33)	24.5 (15–41)		
Fifth year after treatment	Mean (SD)	20.8 (10.5)	25.1 (17.4)	5.02 (1.12, 8.73)	<0.0001
	Median (IQR, Q1–Q3)	20 (11–31)	22 (13–39)		

* *Multivariate model with adjustment for covariates: Age, year of diagnosis, CCI score, myocardial infarction, congestive heart failure, peripheral vascular disease, cerebrovascular disease, chronic pulmonary disease, diabetes, hypertension, income, hospital area, hospital level, clinical T stage, Gleason score, grade, preoperative PSA, and Damico risk classification. Least square mean difference for continuous variables and odds ratio for binary variables through fitting the generalized linear model with stratification of matched pairs*.

*RP, radical prostatectomy; SD, standard deviation; IQR, interquartile range; IMRT, intensity-modulated radiation therapy; Ref, reference group; CI, confidence interval*.

### Hospitalization for Urinary Diseases or Treatment-Related Complications Stratified by RP and IMRT

A significant decrease was observed in the rate of hospitalization for urinary diseases or treatment-related complications for both modalities (Table 2, *p* < 0.05) from the first year (hospitalization rates of 29.96% and 51.83% for RP and IMRT + ADT, respectively, *p* = 0.0013) onward after treatment until the last follow-up (hospitalization rates of 9.80% and 15.97% for RP and IMRT + ADT, respectively, *p* = 0.0379). In the logistic regression multivariate model with adjustment for covariates, the adjusted odds ratio (aOR) (95% CI) of IMRT was 2.10 (1.37–2.56), 1.55 (1.08–2.21), 1.35 (1.08–2.21), 1.24 (1.07–2.21), and 1.09 (1.02–1.81) for the first, second, third, fourth, and fifth years, respectively, compared with that of RP.

**Table 2:** Logistic regression model of hospitalization for urinary diseases or treatment-related complications stratified by RP and IMRT.

**Hospitalization (%)**	**RP (Ref)**, ***N*** **= 277**		**IMRT**, ***N*** **= 382**		aOR (95% CI)*		***p*-value**
	***N*** **, %**		***N*** **, %**				
First year after treatment	83	29.96	198	51.83	2.10	(1.37, 2.56)	0.0013
Second year after treatment	67	24.19	119	31.15	1.55	(1.08, 2.21)	0.0151
Third year after RP	53	19.13	101	26.44	1.35	(1.08, 2.21)	0.0084
Fourth year after treatment	39	14.08	81	21.20	1.24	(1.07, 2.21)	0.0071
Fifth year after treatment	27	9.80	61	15.97	1.09	(1.02, 1.81)	0.0379

* *Multivariate model with adjustment for covariates: Age, year of diagnosis, CCI score, myocardial infarction, congestive heart failure, peripheral vascular disease, cerebrovascular disease, chronic pulmonary disease, diabetes, hypertension, income, hospital area, hospital level, clinical T stage, Gleason score, grade, preoperative PSA, and Damico risk classification. Least square mean difference for continuous variables and odds ratio for binary variables through the fitting generalized linear model with stratification of matched pairs*.

*RP, radical prostatectomy; IMRT, intensity-modulated radiation therapy; Ref, reference group; CI, confidence interval*.

### Medical Reimbursement for Urinary Diseases or Treatment-Related Complications Stratified by RP and IMRT

**Figure 1:**
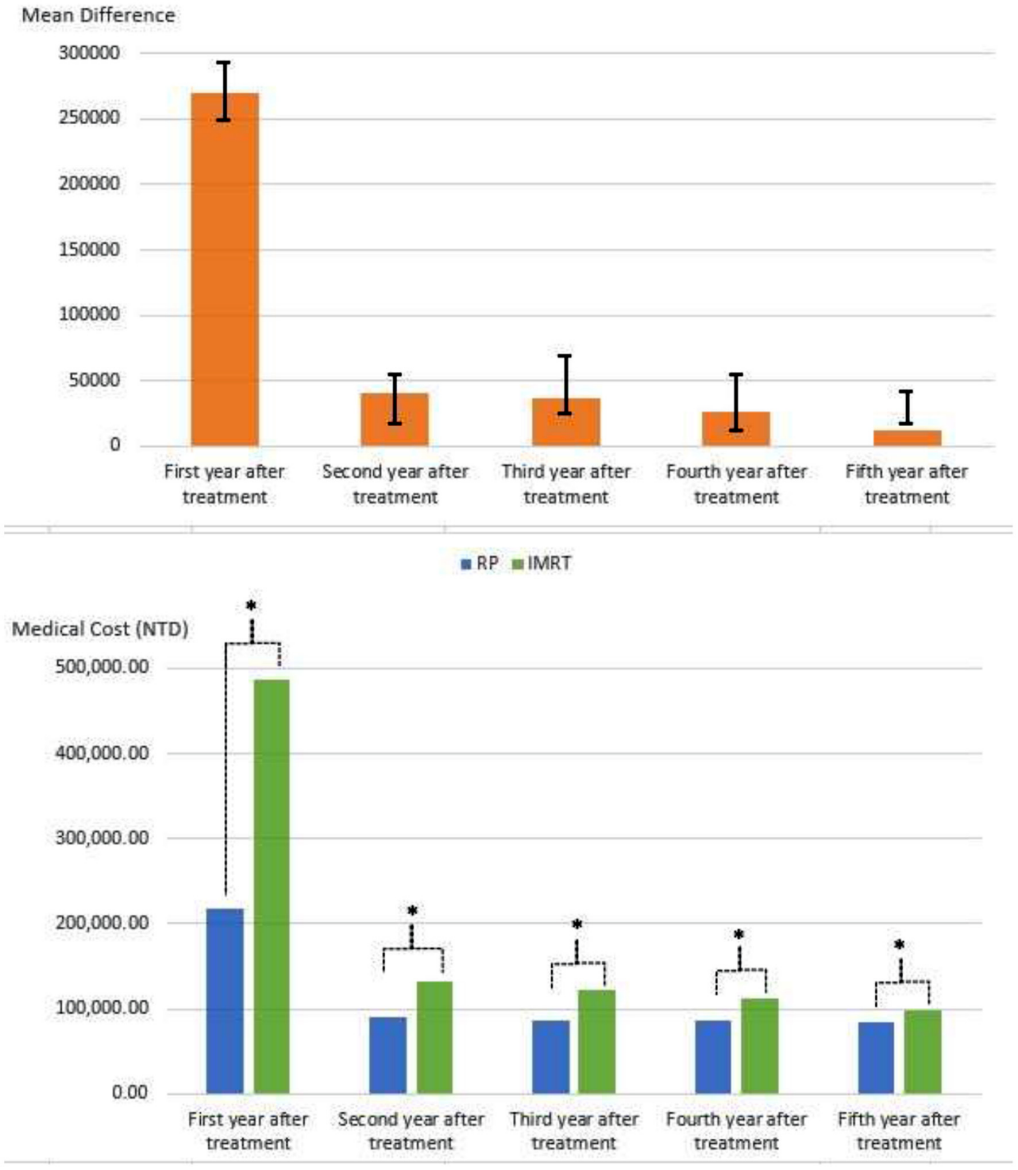
The bar plots of medical costs trends by time stratified by RP and IMRT. RP, radical prostatectomy; IMRT, intensity-modulated radiation therapy; NTD, new Taiwan dollars. *P < 0.05.

Treatment costs were lower for RP because treatment-related complications were fewer after RP than after IMRT, with approximately 55% reduction in the first year (*p* < 0.0001) and ~30% reduction in the second to fourth years (Table 3). The total medical claims amounts of RP and IMRT + ADT over 5 years were 5,65,313 New Taiwan Dollars (NTD) and 9,60,692 NTD in terms of the mean value (Table 3) and 3,51,598 NTD and 7,24,702 NTD in terms of the median value, respectively. RP was associated with a saving of 395,709 NTD, which was approximately 80% of the medical cost of IMRT at that time (Table 3). In the generalized linear mixed model with adjustment for covariates (Supplementary Table 1, online only), the mean difference (95% CI) between RT and IMRT + ADT was 2,69,823 (2,47,676–2,91,970, *p* < 0.0001), 40,803 (17,379–54,228, *p* < 0.0001), 36,202 (24,375–68,029, *p* < 0.0001), 26,708 (11,179–54,595, *p* = 0.0321), and 12,173 (17,140–41,487, *p* < 0.0187) for the first, second, third, fourth, and fifth years, respectively. The bar plots of medical costs trends by time were presented in Figure 1.

**Table 3:** Generalized linear mixed model of medical reimbursement for urinary diseases or treatment-related complications stratified by RP and IMRT.

**Medical cost (NTD)**		**RP (Ref)**, ***N*** **= 277**		**IMRT**, ***N*** **= 382**		Mean difference (95% CI)*	***p*-value**
First year after treatment	Mean (SD)	2,17,606.6	(1,32,341.8)	4,87,430.0	(1,64,201.9)	2,69,823 (2,47,676, 2,91,970)	<0.0001
	Median (IQR, Q1–Q3)	1,76,838	(1,44,113–2,21,615)	4,70,451	(4,25,597–5,23,395)		
Second year after treatment	Mean (SD)	90,479.5	(1,53,852.6)	1,31,282.9	(1,59,277.0)	40,803 (17,379, 54,228)	<0.0001
	Median (IQR, Q1–Q3)	45,563	(2,57,55–77,938)	70,793	(37,366–1,32,908)		
Third year after treatment	Mean (SD)	86,160.1	(1,12,929.6)	1,22,362.0	(1,69,854.7)	36,202 (24,375, 68,029)	<0.0001
	Median (IQR, Q1–Q3)	42,384	(23,572–84,132)	70,088	(34,006–1,36,271)		
Fourth year after treatment	Mean (SD)	85,983.9	(1,74,618.0)	1,12,692.2	(1,75,758.5)	26,708 (11,179, 54,595)	0.0321
	Median (IQR, Q1–Q3)	37,628	(17,125–67,590)	52,330	(23,338–1,26,386)		
Fifth year after treatment	Mean (SD)	85,083.3	(1,30,088.1)	97,256	(1,45,400.2)	12,173 (17,140, 41,487)	0.0187
	Median (IQR, Q1–Q3)	49,185	(28,120–91,347)	61,040	(31,871–1,00,628)		

* *Multivariate model with adjustment for covariates: Age, year of diagnosis, CCI score, myocardial infarction, congestive heart failure, peripheral vascular disease, cerebrovascular disease, chronic pulmonary disease, diabetes, hypertension, income, hospital area, hospital level, clinical T stage, Gleason score, grade, preoperative PSA, and Damico risk classification. Least square mean difference for continuous variables and odds ratio for binary variables through fitting generalized linear model with stratification of matched pairs*.

*RP, radical prostatectomy; SD, standard deviation; IQR, interquartile range; IMRT, intensity-modulated radiation therapy; Ref, reference group; NTD, New Taiwan Dollars; N, number; CI, confidence interval*.

## Discussion

The incidence of LPC is likely to increase in the future due to population aging (the number of older persons is projected to double) and increased life expectancy worldwide (15). In the United States, cancer medical costs showed a 27% increase within 2010–2020, with the largest proportion (42%) of cost accounting for PC (29). In addition, the global cost of PC has increased considerably, from USD11.85 billion in 2010 to USD18.53 billion in 2020 (30). Gaining a comprehensive understanding of therapy cost requires comprehensive knowledge; thus, measuring health care costs is a great challenge faced by health care providers. The society and the national government health departments have prevented such unnecessary expenditure by encouraging bundled payments provided by insurance reimbursement. Such action can systematically reduce the cost throughout PC treatment (30). Nevertheless, no comparative study has evaluated the long-term medical resource consumption of the curative-intended therapies of RP and IMRT for men with HR-LPC until now. To the best of our knowledge, our study is the first population-based study of the long-term medical resource consumption of the RP and IMRT modalities according to the number of urological clinical visits, hospitalization rate, and medical costs for treatment-related complications (Tables 2, 3). According to our findings, RP significantly decreased the number of urology outpatient clinic visits required postoperatively compared with IMRT + ADT and effectively reduced the hospitalization rate for urinary diseases or treatment-related complications as well as succeeding medical reimbursement arise for urinary diseases or treatment-related complications compared with IMRT (Tables 1–3).

From the first year post treatment onward, the number of urology outpatient clinic visits showed a significant difference between the RP and IMRT plus long-term ADT groups (Table 1). The higher number of outpatient clinic visits in the IMRT group indicated the significant medical resource consumption for old men with NCCN-HR-LPC compared with the RP group (median visit numbers of 12.9% and 16.8% for RP and IMRT, respectively, *p* < 0.0001). In each follow-up year (first, second, third, fourth, and fifth years), a higher disparity was observed in the medical resource consumption of the modalities. This finding indicated lower medical resource consumption for old patients with NCCN-HR-LPC who underwent RP than for those who underwent IMRT plus ADT. The number of outpatient clinic visits after 5 years was similar between both modalities, as affected by the slowly regressing medical resource consumption between the RP and IMRT groups. This medical resource consumption (Tables 1–3) might be attributed to treatment-related complications. Side effects after IMRT + ADT include urinary incontinence (31), gastrointestinal toxicity, and soreness and swelling, as after-effects of radiation exposure.

The trend of hospitalization for urinary diseases or treatment-related complications was similar to that of the number of urology outpatient clinic visits (Tables 1, 2). The medical resource consumption of the RP group was superior to that of the IMRT group in the long-term 5-year follow-up due to the fewer hospitalizations (Table 2). With time, the difference in the medical resource consumption between the two treatments narrowed. The hospitalization rates for urinary diseases or treatment-related complications of RP and IMRT in the first and fifth years were 29.96% and 51.83% (*p* = 0.0013) and 9.80% and 15.97% (*p* = 0.0379); thus, in terms of hospitalization, the medical resource consumption of the RP group was half of that of the IMRT group. These findings support the findings of Cooperberg et al. (7) that IMRT is actually less effective and more expensive than RP. The NHI universal program is a compulsory enrollment system for all citizens and foreign residents of Taiwan and thus covers almost the entire population. Up to now, it includes up to 99.8% of the 23.57 million inhabitants of Taiwan. This insurance system ensures that everyone has the same accessibility and affordability to medical care, with the extensive coverage of emergency care, inpatient and outpatient care, imaging and laboratory tests, prescription drugs, traditional Chinese medicine, dental services, and home nursing care (32–35). Therefore, our study represents a comprehensive comparative study of the long-term medical resource consumption of RP and IMRT. Our findings indicate that RP is associated with less medical resource consumption in old men with NCCN-HR-LPC compared with IMRT; this is prevalent even in HR-LPC with more aggressive cancer behavior and advanced tumor stages.

In this study, the generalized linear mixed model was used to evaluate medical reimbursement for urinary diseases or treatment-related complications for RP or IMRT (Table 3). Based on the analysis results, RP was more cost-effective than IMRT in each year or overall from the beginning until the end of the follow-up period. Therefore, this hints that RP has more favorable outcomes with potential fewer complications and side effects, and it has less medical resource consumption. RP is correlated with positive margin rates of up to 50% (36). In addition, the RP approach for NCCN-HR-LPC requires adherence to several principles (37), as follows: (1) complete removal of the gland, (2) confirmed negative surgical margins intraoperatively on the frozen section, and (3) great performance of the extended pelvic lymph node dissection. In general, RP might be more complicated and difficult to perform for advanced tumor stages in men with NCCN-HR-LPC; post-RP complications might be more in men with HR-LPC, especially in old men (38, 39). Our results contradict the hypothesis that old men might be more suitable for IMRT rather than for RP (1, 3, 4). In our results, higher medical resource consumption was found for IMRT than for RP in old men with NCCN-HR-LPC (Tables 1–3). In addition, the mean follow-up time for the two treatments was similar (Supplementary Table 1, online only); therefore, there was no competing risk of mortality in the endpoint of the medical resource consumption between RP and IMRT (40).

RT has several disadvantages, namely time and resource consuming (9, 41). IMRT is the advanced RT technique that enables higher conformal therapy for differentiating the adjacent normal tissue from the targeted tumor, allowing better dose distribution and delivering an escalated dose to the targeted area (9–12). In terms of advantages of IMRT, the cost of IMRT likely depends on radiation conformity, which can decrease the area of tissue exposed to high-dose radiation (9–12). Moreover, the radiation costs are mostly influenced by the total number of treatments and the fixed costs of the equipment (30). With the progression of contemporary RT techniques, the cost of IMRT might be higher in the near future (42, 43). Our study showed a higher medical resource consumption in IMRT than in RP. We believe that the medical resource consumption might be different in the next generation of proton therapy with fewer RT-related complications and toxicities, although the proton therapy is very expensive (42–44).

This study has many strengths. First, the entire dataset of old men with NCCN-HR-LPC undergoing RP and IMRT was retrieved from the TCRD in Taiwan, which represented almost the entire population. Second, the data were collected periodically, and the study population was followed up successively for 5 years. Third, covariates were balanced between the RP and IMRT groups, which decreased bias probability. Additionally, all medical costs have been paid by NHI and data recorded in the NHIRD. Therefore, there is no non-direct costs of care that may confound and/or influence interpretation of these direct costs findings. The findings of this study can assist physicians and patients in choosing the most effective and optimal therapy for old patients with NCCN-HR-LPC considering the medical cost, quality of life, and treatment-related complications. Our findings provide a valuable reference for shared decision-making by old patients and physicians and for establishing health policies for providing national health services. Quality of life and empirical clinical outcomes should be considered when selecting curative-intent treatments in old men with HR-LPC, which are expected to have a higher economic burden in the future, and the most cost-effective treatment option should be determined, especially for HR-LPC. This study provided the first complete nation-wide empirical population-based evidence that RP could be the preferred treatment option for old men with NCCN-HR-LPC considering both clinical and economical endpoints.

However, this study has several limitations. First, it only considered patients with treatment covered by Taiwan's NHI system and did not consider treatment with out-of-pocket payment. However, such old men with NCCN-HR-LPC were most likely to be few. Furthermore, the cost might vary between countries. Therefore, the findings may not be generalized to other countries. Despite these limitations, this is the first population-based cohort study with current updated information and long-term follow-up for the medical resource consumption of RP and IMRT. The results can help in formulating health care policies, particularly for the medical reimbursement of the treatment modalities for the old men with NCCN-HR-LPC.

## Conclusions

The total medical resource consumption in the RP group of old men with NCCN-HR-LPC was less in terms of the number of urology outpatient clinic visits, the number of hospitalizations for urinary diseases or treatment-related complications, and medical reimbursement for urinary diseases or surgical complications compared with the high-dose IMRT plus long-term ADT group.

## Data Availability Statement

The data used in this study is from the National Health Insurance Research Database and Taiwan Cancer Registry Database. The data cannot be made available due to the Personal Information Protection Act executed by Taiwan's government, starting in 2012. Requests for data can be sent as a formal proposal to obtain approval from the Ethics Review committee of the appropriate governmental department in Taiwan. Specifically, links regarding contact info for which data requests may be sent to are as follows: http://nhird.nhri.org.tw/en/Data_Subsets.html#S3 and http://nhis.nhri.org.tw/point.html. Requests to access the datasets should be directed to szuyuanwu5399@gmail.com.

## Ethics Statement

The study protocols were reviewed and approved by the Institutional Review Board of Tzu-Chi Medical Foundation (IRB109-015-B). Written informed consent for participation was not required for this study in accordance with the national legislation and the institutional requirements.

## Author Contributions

S-YW, FE, JP, and C-CH: conception, design, and manuscript writing. S-YW and C-CH: collection and assembly of data. S-YW: data analysis, interpretation, and administrative support. All authors: final approval of manuscript.

## Funding

This work was supported by Lo-Hsu Medical Foundation, Lotung Poh-Ai Hospital, supports S-YW work (Funding Numbers: 10908, 10909, 11001, 11002, 11003, 11006, and 11013).

## Conflict of Interest

FE was employed by PT Inertia Utama. JP was employed by Roche Diagnostics Ltd. The remaining authors declare that the research was conducted in the absence of any commercial or financial relationships that could be construed as a potential conflict of interest.

## Publisher's Note

All claims expressed in this article are solely those of the authors and do not necessarily represent those of their affiliated organizations, or those of the publisher, the editors and the reviewers. Any product that may be evaluated in this article, or claim that may be made by its manufacturer, is not guaranteed or endorsed by the publisher.

## Abbreviations

PC: Prostate cancer
HR-LPC: high-risk localized prostate cancer
RP: radical prostatectomy
RT: radiotherapy
IMRT: intensity-modulated radiotherapy
aOR: adjusted odds ratio
OR: odds ratio
CI: confidence interval
NCCN: National Comprehensive Cancer Network
PSA: prostate-specific antigen
NCCN-HR-LPC: National Comprehensive Cancer Network high-risk localized prostate cancer
PSM: propensity score matching
NHIRD: National Health Insurance Research Database
TCRD: Taiwan Cancer Registry Database
AJCC: American Joint Committee on Cancer
ADT: androgen deprivation therapy
ICD-9-CM: International Classification of Diseases, Ninth Revision, Clinical Modification
CCI: Charlson comorbidity index
T: tumor
SD: standard deviation
RARP: robot-assisted radical prostatectomy
NHI: National Health Insurance
NTD: New Taiwan Dollars
US: United States
USD: United States Dollars.
