# Supplementary material for: Long-Term Medical Resource Consumption of Radical Prostatectomy vs. Intensity-Modulated Radiotherapy for Old Patients With Prostate Cancer: A Nationwide Population-Based Cohort Study
Source: Front Med (Lausanne). 2022 May 3;9:843709. doi: 10.3389/fmed.2022.843709 (PMC9113182; doi:10.3389/fmed.2022.843709)
Supplement: Supplementary file 1 [file Table_1.pdf]

TABLE S1. Characteristics of older men aged ≥80 years with high-risk prostate adenocarcinoma stratified by whether they underwent RP or IMRT+HT

Covariates		RP N = 277 n	(%)	IMRT + long-term HT N = 382 n	(%)	*P* value
Age	Mean (SD)	84.6	(3.2)	85.1	(3.5)	.1098
	Median (Q1–Q3)	84	(82–86)	85	(82–87)	
	80–89	256	(92.4)	340	(89.0)	.9090
	90+	21	(7.6)	42	(11.0)	
Year of diagnosis	2011–2012	47	(17.0)	60	(15.7)	.9837
	2013	49	(17.7)	72	(18.8)	
	2014	57	(20.6)	82	(21.5)	
	2015	62	(22.4)	83	(21.7)	
	2016	62	(22.4)	85	(22.3)	
CCI scores	0	102	(36.8)	145	(38.0)	.7626
	1	77	(27.8)	104	(27.2)	
	2+	98	(35.4)	133	(34.8)	
Myocardial infarction		4	(1.4)	7	(1.8)	.8348
Congestive heart failure		10	(3.6)	13	(3.4)	.7740
Peripheral vascular disease		10	(3.6)	10	(2.6)	.4913
Cerebrovascular disease		31	(11.2)	48	(12.6)	.8997
Chronic pulmonary disease		41	(14.8)	57	(14.9)	.7090
Diabetes		73	(26.4)	94	(24.6)	.6732

Covariates		RP N = 277 n	(%)	IMRT + long-term HT N = 382 n	(%)	*P* value
Hypertension		166	(59.9)	233	(61.0)	.9782
Income	<NTD 21,000	92	(33.2)	119	(31.2)	.8053
	NTD 21,000–30,000	124	(44.8)	170	(44.5)	
	NTD 30,000–45,000	29	(10.5)	41	(10.7)	
	NTD 45,000+	32	(11.6)	52	(13.6)	
Hospital area	North	128	(46.2)	169	(44.2)	.9637
	Central	82	(29.6)	115	(30.1)	
	South	61	(22.0)	89	(23.3)	
	East	6	(2.2)	9	(2.4)	
Hospital level	Medical center	178	(64.3)	244	(63.9)	.5094
	others	99	(35.7)	138	(36.1)	
Clinical T stage	T1	96	(34.7)	128	(33.5)	.9366
	T2a	57	(20.6)	77	(20.2)	
	T2b	26	(9.4)	44	(11.5)	
	T2c	89	(32.1)	120	(31.4)	
	T3a	9	(3.2)	13	(3.4)	
Gleason score	≤5	0		0		.9570
	6	19	(6.9)	33	(8.6)	
	7	187	(67.5)	253	(66.2)	
	8	44	(15.9)	64	(16.8)	
	9+	22	(7.9)	23	(6.0)	

Covariates		RP N = 277 n	(%)	IMRT + long-term HT N = 382 n	(%)	*P* value
	Missing	5	(1.8)	9	(2.4)	
Grade (max Gleason grade)	1-2	5	(1.8)	9	(2.4)	.9624
	3	19	(6.9)	32	(8.4)	
	4	223	(80.5)	304	(79.6)	
	5	30	(10.8)	37	(9.7)	
Preoperative PSA, ng/mL	Mean (SD)	11.7	(7.6)	12.7	(11.6)	.1942
	Median (IQR)	10.3	(6.8–15.1)	10.5	(7.2–14.9)	
	0–5	22	(7.9)	34	(8.9)	.9770
	5–10	95	(34.3)	116	(30.4)	
	10–20	109	(39.4)	158	(41.4)	
	20+	15	(5.4)	28	(7.3)	
	Missing	36	(13.0)	46	(12.0)	
D'Amico risk classification	Localized intermediate	125	(45.1)	171	(44.8)	.8449
	Localized high	140	(50.5)	194	(50.8)	
	Localized advanced	12	(4.3)	17	(4.5)	
Follow-up time, months	Mean (SD)	61.7	(18.4)	58.4	(18.9)	.8742

RP, radical prostatectomy; IMRT, intensity-modulated radiation therapy; IQR, interquartile range; SD, standard deviation; T, tumor; PSA, prostate-specific antigen; N, number; HT, hormone therapy; NTD, new Taiwan dollars
